# Male Circumcision at Different Ages in Rwanda: A Cost-Effectiveness Study

**DOI:** 10.1371/journal.pmed.1000211

**Published:** 2010-01-19

**Authors:** Agnes Binagwaho, Elisabetta Pegurri, Jane Muita, Stefano Bertozzi

**Affiliations:** 1 Ministry of Health, Kigali, Rwanda; 2 UNAIDS, Kigali, Rwanda; 3 UNICEF, Kigali, Rwanda; 4 Centro de Investigación Evaluación y Encuestas, Instituto Nacional de Salud Pública, Cuernavaca, Morelos, Mexico; University of Connecticut, United States of America

## Abstract

**Background:**

There is strong evidence showing that male circumcision (MC) reduces HIV infection and other sexually transmitted infections (STIs). In Rwanda, where adult HIV prevalence is 3%, MC is not a traditional practice. The Rwanda National AIDS Commission modelled cost and effects of MC at different ages to inform policy and programmatic decisions in relation to introducing MC. This study was necessary because the MC debate in Southern Africa has focused primarily on MC for adults. Further, this is the first time, to our knowledge, that a cost-effectiveness study on MC has been carried out in a country where HIV prevalence is below 5%.

**Methods and Findings:**

A cost-effectiveness model was developed and applied to three hypothetical cohorts in Rwanda: newborns, adolescents, and adult men. Effectiveness was defined as the number of HIV infections averted, and was calculated as the product of the number of people susceptible to HIV infection in the cohort, the HIV incidence rate at different ages, and the protective effect of MC; discounted back to the year of circumcision and summed over the life expectancy of the circumcised person. Direct costs were based on interviews with experienced health care providers to determine inputs involved in the procedure (from consumables to staff time) and related prices. Other costs included training, patient counselling, treatment of adverse events, and promotion campaigns, and they were adjusted for the averted lifetime cost of health care (antiretroviral therapy [ART], opportunistic infection [OI], laboratory tests). One-way sensitivity analysis was performed by varying the main inputs of the model, and thresholds were calculated at which each intervention is no longer cost-saving and at which an intervention costs more than one gross domestic product (GDP) per capita per life-year gained. Results: Neonatal MC is less expensive than adolescent and adult MC (US$15 instead of US$59 per procedure) and is cost-saving (the cost-effectiveness ratio is negative), even though savings from infant circumcision will be realized later in time. The cost per infection averted is US$3,932 for adolescent MC and US$4,949 for adult MC. Results for infant MC appear robust. Infant MC remains highly cost-effective across a reasonable range of variation in the base case scenario. Adolescent MC is highly cost-effective for the base case scenario but this high cost-effectiveness is not robust to small changes in the input variables. Adult MC is neither cost-saving nor highly cost-effective when considering only the direct benefit for the circumcised man.

**Conclusions:**

The study suggests that Rwanda should be simultaneously scaling up circumcision across a broad range of age groups, with high priority to the very young. Infant MC can be integrated into existing health services (i.e., neonatal visits and vaccination sessions) and over time has better potential than adolescent and adult circumcision to achieve the very high coverage of the population required for maximal reduction of HIV incidence. In the presence of infant MC, adolescent and adult MC would evolve into a “catch-up” campaign that would be needed at the start of the program but would eventually become superfluous.

*Please see later in the article for the Editors' Summary*

## Abstract 2

Agnes Binagwaho and colleagues predict that circumcision of newborn boys would be effective and cost-saving as a long-term strategy to prevent HIV in Rwanda.

## Editors' Summary

**Background:**

Acquired immunodeficiency syndrome (AIDS) has killed more than 25 million people since 1981 and more than 31 million people (22 million in sub-Saharan Africa alone) are now infected with the human immunodeficiency virus (HIV), which causes AIDS. There is no cure for HIV/AIDS and no vaccine against HIV infection. Consequently, prevention of HIV transmission is extremely important. HIV is most often spread through unprotected sex with an infected partner. Individuals can reduce their risk of HIV infection, therefore, by abstaining from sex, by having one or a few sexual partners, and by always using a male or female condom. In addition, male circumcision—the removal of the foreskin, the loose fold of skin that covers the head of penis—can halve HIV transmission rates to men resulting from sex with women. Thus, as part of its HIV prevention strategy, the World Health Organization (WHO) recommends that male circumcision programs be scaled up in countries where there is a generalized HIV epidemic and where few men are circumcised.

**Why Was This Study Done?:**

One such country is Rwanda. Here, 3% of the adult population is infected with HIV but only 15% of men are circumcised—worldwide, about 30% of men are circumcised. Demand for circumcision is increasing in Rwanda but, before policy makers introduce a country-wide male circumcision program, they need to identify the most cost-effective way to increase circumcision rates. In particular, they need to decide the age at which circumcision should be offered. Circumcision soon after birth (neonatal circumcision) is quick and simple and rarely causes any complications. Circumcision of adolescents and adults is more complex and has a higher complication rate. Although several studies have investigated the cost-effectiveness (the balance between the clinical and financial costs of a medical intervention and its benefits) of circumcision in adult men, little is known about its cost-effectiveness in newborn boys. In this study, which is one of several studies on male circumcision being organized by the National AIDS Control Commission in Rwanda, the researchers model the cost-effectiveness of circumcision at different ages.

**What Did the Researchers Do and Find?:**

The researchers developed a simple cost-effectiveness model and applied it to three hypothetical groups of Rwandans: newborn boys, adolescent boys, and adult men. For their model, the researchers calculated the effectiveness of male circumcision (the number of HIV infections averted) by estimating the reduction in the annual number of new HIV infections over time. They obtained estimates of the costs of circumcision (including the costs of consumables, staff time, and treatment of complications) from health care providers and adjusted these costs for the money saved through not needing to treat HIV in males in whom circumcision prevented infection. Using their model, the researchers estimate that each neonatal male circumcision would cost US$15 whereas each adolescent or adult male circumcision would cost US$59. Neonatal male circumcision, they report, would be cost-saving. That is, over a lifetime, neonatal male circumcision would save more money than it costs. Finally, using the WHO definition of cost-effectiveness (for a cost-effective intervention, the additional cost incurred to gain one year of life must be less than a country's per capita gross domestic product), the researchers estimate that, although adolescent circumcision would be highly cost-effective, circumcision of adult men would only be potentially cost-effective (but would likely prove cost-effective if the additional infections that would occur from men to their partners without a circumcision program were also taken into account).

**What Do These Findings Mean?:**

As with all modeling studies, the accuracy of these findings depends on the many assumptions included in the model. However, the findings suggest that male circumcision for infants for the prevention of HIV infection later in life is highly cost-effective and likely to be cost-saving and that circumcision for adolescents is cost-effective. The researchers suggest, therefore, that policy makers in Rwanda and in countries with similar HIV infection and circumcision rates should scale up male circumcision programs across all age groups, with high priority being given to the very young. If infants are routinely circumcised, they suggest, circumcision of adolescent and adult males would become a “catch-up” campaign that would be needed at the start of the program but that would become superfluous over time. Such an approach would represent a switch from managing the HIV epidemic as an emergency towards focusing on sustainable, long-term solutions to this major public-health problem.

**Additional Information:**

Please access these Web sites via the online version of this summary at http://dx.doi.org/10.1371/journal.pmed.1000211.

- This study is further discussed in a *PLoS Medicine* Perspective by Seth Kalichman
- Information is available from the US National Institute of Allergy and Infectious Diseases on HIV infection and AIDS
- Information is available from the Joint United Nations Programme on HIV/AIDS (UNAIDS) on HIV infection and AIDS and on male circumcision in relation to HIV and AIDS
- HIV InSite has comprehensive information on all aspects of HIV/AIDS
- Information is available from Avert, an international AIDS charity on many aspects of HIV/AIDS, including information on HIV and AIDS in Africa, and on circumcision and HIV (some information in English and Spanish)
- More information about male circumcision is available from the Clearinghouse on Male Circumcision
- The National AIDS Control Commission of Rwanda provides detailed information about HIV/AIDS in Rwanda (in English and French)

## Introduction

Male circumcision (MC) is one of the oldest and most common surgical procedures with approximately 30% of men circumcised worldwide [1]. While MC is almost universal in North Africa and most of West Africa, it is less common in Southern Africa where HIV prevalence is much higher. In Rwanda, MC is not a traditional procedure and it is estimated that only about 15% [2] of men are circumcised. Nonetheless, due to the ongoing debate about MC in the country, demand for the service is increasing (Ministry of Health [MOH], Rwanda).

Conclusive evidence from three randomised control trials conducted in Uganda, Kenya, and South Africa showed that MC reduces the risk of HIV infection by about 55%: 51% in Uganda [3], 53% in Kenya [4], and 60% in South Africa [5].

Studies also report a substantially reduced risk of other sexually transmitted infections (STIs) among circumcised men, such as syphilis (summary risk ratio [RR] = 0.67, 95% confidence interval [CI] 0.54–0.83) and chancroid (RR 0.12–1.11) [6]. In these studies, the reduced risk of herpes simplex virus type 2 (HSV-2) infection is of borderline statistical significance (summary RR = 0.88, 95% CI 0.77–1.01) [6]; however, a recent study shows that MC significantly reduces the incidence of HSV-2 (adjusted RR 0.72, 95% CI 0.56–0.92; *p* = 0.008) [7]. There is also evidence that MC protects against urinary tract infections (RR = 0.13, CI 0.01–2.63) [8]; invasive penile cancer [9]; and reduces prevalence of human papillomavirus (HPV) (adjusted RR 0.65, 95% CI 0.46–0.90; *p* = 0.009) [7]. Most studies report a reduced risk of gonorrhoea and chlamydia trachomatis infection in female partners [1]. Moreover, MC protects against balanitis, posthitis, phimosis, and paraphimosis [9].

MC can be performed at different ages, with important differences in complication rates. Neonatal circumcision is a simple, quick procedure, healing within 1 wk with a low rate of usually minor adverse events (0.2%–0.4% in the US) when performed in clinical settings by trained professionals [10]. In adults the procedure is more complex and usually takes 4–6 wk for the wound to fully heal. The rate of complications (such as pain, bleeding, infections, and/or injury) ranges from 2% to 4% when performed under optimal conditions [10].

Adult HIV prevalence in Rwanda is 3.0% (95% CI 2.6–3.4) [11] with HIV transmission mainly occurring through heterosexual sex. Prevalence among men is 2.3%; 3.8% among circumcised men and 2.1% among uncircumcised men [11]. However, when comparing men living in urban areas, where seroprevalence is higher and where the majority of circumcised men live, prevalence among circumcised men (5.0%) is lower than among uncircumcised men (5.7%) [11]. On the basis of these data and particularly given the conclusive evidence in favour of MC in the randomised trials cited, the National AIDS Commission of Rwanda (CNLS) developed a cost-effectiveness model in order to better inform policy and programmatic decisions about implementing a MC program in the country. Complementary studies on knowledge, attitudes, and practice of MC are ongoing.

A cost-effectiveness study on MC for infants and adolescents is needed given the fact that the MC debate in Southern Africa has focused primarily on MC for adults. Without a vaccine or cure for AIDS available, the CNLS in Rwanda felt that strategic planning of interventions should take a longer-term perspective and include future generations. We hypothesize that a strategy combining infant and adult/adolescent circumcision would be both more cost-effective and more sustainable than circumcising only adult men. This is the first time, to our knowledge, that a cost-effectiveness study on MC has been carried out in a country where HIV prevalence is below 5% [12]–[16].

## Methods

The analysis adopts the perspective of the Government of Rwanda as a health care payer. In the absence of available tools to evaluate the impact of neonatal, adolescent, and adult MC, a basic cost-effectiveness model was developed. Calculations refer to an average Rwandan adolescent or adult male, and reflect risk factors for HIV such as age at first intercourse and presence of STIs, as well as sexual behaviours such as condom use and number/concurrency of partners.

Effectiveness, defined as the number of HIV infections averted, was calculated by projecting the reduction in HIV incidence over time. Costs included the materials necessary for performing circumcisions, staff time, associated staff training, patient counselling, the treatment of adverse events, and related promotion campaigns, and were adjusted for the averted lifetime cost of health care (antiretroviral therapy [ART], opportunistic infections [OIs], laboratory tests), conservatively considering only averted HIV treatment costs, not those of other STIs.

One-way sensitivity analysis was performed by varying the main inputs of the model, and the thresholds at which each intervention (a) is no longer cost-saving and (b) costs more than one GDP per capita per life-year gained were calculated for the following variables: discount rate, HIV incidence, protection rate of MC, the cost of MC, the cost of health care averted, and adherence to ART.

The model was applied to three hypothetical male cohorts in Rwanda in 2008: newborns, adolescents, and adult men. The number of male infants born in Rwanda in 2008 is estimated to be approximately 210,000 (Rwanda National Institute of Statistics [RNIS], 2009). Although only 38% of births occur in health facilities [11], 97% of newborns receive bacille Calmette-Guérin vaccination in a health facility within 1 mo of birth (Vaccination programme Rwanda/PEV, December 2007). This visit to a health facility provides an opportunity to circumcise the infant, thus making it feasible to offer circumcision to nearly all infants, of which we estimate at least 70% are likely to undergo the procedure. Acceptance of MC in Rwanda is expected to be high since there are no cultural barriers to it, demand is already on the rise (MOH, Rwanda), and the intervention is expected to be accompanied by an intense national promotion campaign. The numbers of circumcisions would be about 150,000 children annually. To facilitate comparisons for this exercise, we considered a similarly sized cohort of adolescents and of adults (there are 2,140,000 males older than 15 y in the country, RNIS), although optimal implementation strategies should probably aim for higher annual coverage of adolescents and adults during the initial years of the program.

For purposes of modelling, we assumed that infants are circumcised at birth, adolescents at age 15 y, and adults at age 30 y. The model projects HIV infections averted until death. The average life expectancy in Rwanda is 52 y at birth, 62 y at age 15 y, and 64 y at age 30 y [17].

### Effectiveness

Effectiveness is the product of the number of people susceptible to HIV infection in the cohort, the HIV incidence rate at different ages, and the protective effect of MC; discounted back to the year of circumcision and summed over the life expectancy of the circumcised men.

#### People susceptible to HIV infection in the cohort

The analysis of effectiveness is limited to those adolescents and adults who are HIV negative. The model includes the cost of voluntary HIV testing and counselling (VCT) for all adolescents and adults. In keeping with current UNAIDS recommendations [18], MC will be offered regardless of HIV status and of whether a client accepts VCT. MC would only be withheld if it is medically contraindicated. For HIV prevalence, we used the rate reported in the 2005 Rwandan Demographic and Health Survey (RDHS 2005) (0.4% for 15 y olds and 4.2% for 30-y-old men). Given the high coverage of prevention of mother to child transmission (PMTCT) programs in Rwanda (72% of pregnant women in need of PMTCT services have access to them and more than 60% of all health facilities in the country provided PMTCT services in 2008) and the Universal Access targets the country has set for the next few years (90% coverage by 2012) [19], we expect the proportion of children born HIV positive to become negligible over time. Therefore, effectiveness of MC for children is extended to the entire cohort.

#### Estimation of age-specific incidence rates

HIV incidence in Rwanda in 2008 per 5-y age groups (15–19, 20–24, 25–29, 30–34, 35–39, 40–44, 45–49) was estimated using the Estimation and Projection Package (EPP) and Spectrum software, developed by UNAIDS and the Futures Group under the USAID Health Policy Initiative. EPP and Spectrum are a suite of mathematical models based on demographic (RNIS), epidemiological (RDHS 2005, sentinel surveillance), and programmatic (ART and PMTCT, MOH) data that are used for official HIV estimates in Rwanda. Within the age groups, incidence was assumed as equal at each age point. For instance, HIV incidence at 30 y of age was calculated as total incidence for the 30–34 age group divided by 5. Total incidence among men over the age of 15 y is assumed to be due to insertive sexual intercourse. The infections averted for infants are conservatively estimated because they do not include any infections averted prior to age 15 y, as no data are available regarding sexually acquired infections prior to age 15 y.

#### Calculation of cumulative incidence

The future, age-specific, annual HIV incidence was assumed to remain constant at the 2008 rate. To account for the uncertainty related to this assumption we varied the HIV incidence rate during sensitivity analysis. We calculated the probability for each age cohort of becoming infected with HIV over their remaining years of life (cumulative incidence) as the probability of getting infected in year *x*_0_; added to the probability of getting infected in year *x*_0_+*1* = *x*_1_ if not already infected in year *x*_0_ and so on.

#### Discounting health effects

Because health effects occur several years in the future, following common practice in cost-effectiveness analysis, we applied an annual discount rate of 3% [20]. Incidence discounted to 2008 was calculated as: incidence at age *x*/(1+3%)^age *x*^ and summed over each of the 5-y age periods. Since a 3% discount rate may be low for Rwanda, higher discount rates were used in the sensitivity analysis.

#### Efficacy of MC

In accordance with the randomised control trials on MC and HIV prevention previously cited, we used an average value of 55% for the protective effect of MC and assumed that it was constant over the lifetime of the individual.

#### Calculation of infections averted

Infections averted were calculated as HIV incidence over the 5-y age groups multiplied per the size of the cohort and per the efficacy of MC (55%). People already HIV positive at age 15 y and at age 30 y received HIV testing and counselling, but were not circumcised (150,000×[1−HIV prevalence at age 15 or 30 y]). The future stream of infections averted were discounted back to 2008.

#### Calculation of years of life saved

On average, the time between HIV infection and needing treatment is 8 y [21]. For per-person years of survival under treatment we referred to the average life expectancy under care as reported by the US-based Walensky study corresponding to a time in the US before treatment of patients with multidrug resistance was available (and approximating the situation in Rwanda today) [22]. This number corresponds to a life expectancy of 14.9 y without discounting (in the absence of treatment, it would only be 1.6 y from AIDS diagnosis). In order to account for conditions in Rwanda, which are less favourable than they were in the US (such as lower access to second-line therapies and higher rates of competing mortality from other causes, among others), we decreased life expectancy to 14 y on average for the base case analysis. The uncertainty of this value is accounted for during sensitivity analysis. Thus, life years saved are those that are lost from 8+14 = 22 y following infection until that person's life expectancy at the age of infection.

Table 1 provides a summary of the variables used to calculate effectiveness of MC in Rwanda and shows incidence (new infections) among men per age group.

**Table 1:** Effectiveness of neonatal, adolescent, and adult MC in Rwanda, 2008.

Subgroup	Variables	Values							Total
	**Age groups (y)**	15–19	20–24	25–29	30–34	35–39	40–44	45–49	—
	**Incidence rate (cumulative over the age group) (Spectrum)**	0.04%	0.08%	0.36%	0.37%	0.27%	0.33%	0.12%	1.56%
**Infants (born in 2008)**	**Projection period**	2023–27	2028–32	2033–37	2038–42	2043–47	2048–52	2053–57	—
	**Averted infections in the cohort** a	31	66	294	307	222	271	97	1,288
	**Discounted averted infections**	19	35	132	119	74	78	24	482
**Adolescents**	**Projection period**	2008–12	2013–17	2018–22	2023–27	2028–32	2033–37	2038–42	—
	**Averted infections**	31	66	293	305	221	270	97	1,283
	**Discounted averted infections**	29	54	205	185	115	122	38	748
**Adults**	**Projection period**	—	—	—	2008–12	2013–17	2018–22	2023–27	—
	**Averted infections**	—	—	—	294	213	260	93	859
	**Discounted averted infections**	—	—	—	277	173	182	56	689

a The incidence rates are multiplied by cohorts of 150,000, minus the number of infections that occurred previously.

### Costs

Direct costs were modelled on the basis of interviews with experienced health care providers and MOH officials in Kigali to determine all inputs involved in a procedure (from staff time to consumables) and related prices. Health care providers were asked to base their estimates on actual cases they participated in. The validity of the costing model was counterchecked with recently published World Health Organization (WHO) and UNAIDS guidelines/protocols [10].

For infants, we estimated the cost of circumcision employing the Mogen Clamp method. The Mogen Clamp method was chosen because it is a simple procedure that requires only one reusable piece, does not require suturing, and causes less pain and complications than other methods, though there is a risk of injury if not applied carefully [10]. This method appears suitable for national roll-out, even in remote areas.

Since the national HIV policy in Rwanda discourages vertical programs and strongly promotes integration into existing services, infant MC would be integrated into existing neonatal and vaccination services and we expect no cost for infrastructure development. Although the procedure would be integrated into health facilities' existing services, the complexity, time, and space involved in adult MC will require infrastructure investment. Hence, to circumcise 150,000 adults we accounted for 94 additional small surgical rooms (eight procedures/day/room per 200 d/year) with a 10-y useful life. The Central Purchasing of Essential Medicines in Rwanda (CAMERWA) and private pharmacies in Kigali provided wholesale price quotations for consumables.

Unit costs from a recent costing exercise carried out in Rwanda [23] were used for nonmedical inputs such as the implementation of a nationwide promotion campaign. Additional budgeting information from current practice in the health system was used for calculating the cost of training staff and counselling of patients.

Costs of complications were based on calculations from a recent publication on cost-effectiveness of adult MC in South Africa [15], using findings from the Orange Farm MC Trial. Overall cost of adverse events standardised to one person is US$1.03. For children we used half of this amount (US$0.50 per MC). Given that the frequency of side effects in children is less than half the adult rate, and the average complication less severe, this is a conservative estimate.

In the case of adolescents and adults we added the incremental cost of testing and counselling for HIV (US$9.29) [24]. This value compares well with average costs in other African countries [23] and does not include fixed costs of installing new VCT centres. This last assumption is due to the fact that HIV tests are already offered as an integrated service in Rwanda and are widely available in the existing health centres.

A summary of costs is provided in Table 2. Details of unit costs are available from the authors upon request. The higher cost of MC in adolescents and adults is due to several reasons including the higher cost of imported consumables involved in this more complex surgical procedure (the single most expensive item being local anaesthetic), laboratory tests, amortization costs for the surgical kits, the cost of HIV testing and counselling, the increased staff and staff time necessary, and the need for infrastructure scale-up. As expected, for both children and adolescents/adults, the costs of performing MC are below the prices currently charged by private practitioners.

**Table 2:** Costs standardised to one MC procedure for a cohort of 150,000 adults and 150,000 newborns in Rwanda, 2008.

Costs (Direct Costs of Procedure)	Unit Cost Infant MC (US$)	Unit Cost Adolescent/Adult MC (US$)
**Mogen Clamp (reusable 1,000 times)**	0.3	NA
**Infant consumables: 1 g EMLA cream; two impregnated gauze; one absorbable suture; 100 ml antiseptic; 1 ml glucose solution; two pairs gloves)**	6.5	NA
**Adolescent/adult consumables: lidocaine anaesthetic, 15 ml; suture material, three; gauze, 15; sticking plaster; anti-inflammatory; two syringes and needles; antiseptic; gloves masks; caps and aprons; condoms**	NA	21
**Surgery kit, including sterilization costs and amortization**	0.6	8
**Laboratory exams (bleeding time, full blood count)**	—	6
**Staff time infant: nurse A1 level (30 min plus counselling), public system**	2.5	NA
**Staff time adolescent/adult: surgeon and nurse (1 h plus counselling), public system plus 15 min bandage three times every 5 d**	NA	6
**Programme costs**		
**Promotion campaign (over a cohort of 150 000 people)** a	3.5	3.5
**Training of health professionals, including counselling and supervision**	0.8	1.3
**Infrastructure costs**		
**94 small surgical rooms (adolescent/adult)**	NA	2.8
**Side effects**	0.5	1
**Subtotal**	15	50
**Cost of HIV testing and counselling**	—	9.2
**TOTAL (rounded amounts)**	15	59
**Prices paid by clients, Kigali**		
**Health centre**	starting 13^b^ (MOH standard)	—
**Private sector**	45^b^	87

Base case year, 2008.

a The costs of a promotion campaign for a circumcision program that targeted infants, adolescent, and adults would be less than the sum of the individual campaigns, which would increase the cost-effectiveness of each if implemented jointly.

b Dorsal slit method (reflecting current practice in Kigali) in hospital.

These cost estimates do not include the possibility of large economies of scale, for instance those resulting from large orders of supplies and equipment, or cost reduction through the judicious use of task shifting to nurses/health workers. Since we do not know to what level of scale economies might be attained, we account for the possibility of lower unit costs in the context of a large-scale MC program in the sensitivity analysis.

#### Savings

Savings correspond to the lifetime costs of HIV treatment for the HIV infections averted (average number of years of survival under treatment multiplied by the annual cost of treatment and care), adjusted for the rate of access to treatment and adherence.

#### Cost of treatment

Costs of AIDS treatment and care include ART (first and second line), treatment for major OIs, laboratory tests, and home-based care. Average unit costs from a recent costing exercise carried out in Rwanda [23] were used. Primary sources for this compilation exercise included the MOH, TRAC Plus CIDC, CAMERWA, National Reference Laboratory, AEDS, WHO/INSP, the Clinton Foundation, and health services providers such as ARBEF. Table 3 provides a base case summary of treatment and care savings per HIV infection averted. Values are similar to those reported in the international literature for neighbouring countries and studies in Rwanda [25].

**Table 3:** Life-time savings per each HIV infection averted.

Category of Savings per Each HIV Infection Averted	Input Values and Assumptions	Life-Time Costs per Person/Amounts over 14 y (US$ 2008 )
**Home-based care**	US$40 per year for counselling, medical care, clothes, nutrition, etc..). 10% of symptomatic PLHIV receive home-based care in Rwanda.^a^	56
**Prophylaxis and treatment of major OIs**	Average 3 y per person. US$160 is the WHO average treatment cost for sub-Saharan Africa. Cost of generic for co-trimoxazole, fluconazol, anti-TB drugs is US$122 (CAMERWA).	846
**Laboratory tests including supplies**	70% of patients. Average cost is US$73 per year per person (CD4, US$8; viral load, US$25; DNA PCR US$15; hematology, US$6; biochemistry, US$20, including reagents and all supplies plus annualized cost of equipment), National Laboratory.	715
**ART**	ART per person per year first line is $710 (80% of patients). TB per year is $201 (8%), TB costs over 2 y. Cost of therapy given for toxicity (to treat secondary effects, pregnancy, etc.) is $402 (15% patients per 1 y). Cost of second-line therapy is $1,726 (20% of patients). Palliative care is $267 per life-time (10% of patients).	12,890
**Total**	Rounded amounts	14,500
	90% of the cohort (to account for losses to follow-up)	13,050

a Coverage assumptions come from published literature (WHO) and routine data/consensus discussions among HIV service providers in Rwanda.

PLHIV, people living with HIV/AIDS; TB, tuberculosis.

#### Cohort to which apply savings

AIDS treatment costs apply to 90% of the subcohort of people living with HIV. This percentage was obtained assuming 95% access to ART (a realistic assumption for Rwanda given an estimated coverage rate of 80% in 2008 and the ambitious targets for scale-up [19].) and considering the current high level of adherence to treatment and low losses to follow-up [26]. Given the inevitable uncertainty related to assumptions about future care, we varied overall cost (depending on coverage and cost of treatment) during sensitivity analysis. We also specifically varied the values of adherence to treatment.

#### Discounting savings

To calculate discounted savings, treatment costs were multiplied by the number of discounted infections averted and then discounted for the delay from infection averted to averted treatment costs (8 y plus two-thirds of 14 y average survival on treatment). Since HIV infections averted were already discounted back to time of circumcision, we discounted treatment costs only back to the time of the infections averted to avoid double discounting. Treatment costs were discounted using the same rate used for effects and as if they happened at two-thirds between the average age at which treatment starts and death. This assumption takes into account that ART costs are back-loaded and most OI-related costs and home-based care occur in the years prior to death. Table 4 presents savings and discounted savings for the three cohorts in Rwanda.

**Table 4:** Savings for neonatal, adolescent, and adult MC in Rwanda, 2008.

Subgroup	Variables	Values							Total
	**Age groups**	15–19	20–24	25–29	30–34	35–39	40–44	45–49	—
**Infants**	**Projection period**	2023–27	2028–32	2033–37	2038–42	2043–47	2048–52	2053–57	—
	**Averted infections in the cohort**	31	66	294	307	222	271	97	1,288
	**Savings in US$ = lifetime cost of treatment per person × 90% adherence × averted infections**	404,550	861,300	3,836,700	4,006,350	2,897,100	3,536,550	1,265,850	16,808,400
	**Discounted averted infections**	19	35	132	119	74	78	24	482
	**Discounted savings US$ = (lifetime cost of treatment per person × 90% adherence × discounted averted infections)/([1 + 3%]^delay^). Delay = (8 + 2/3 × ** [**14**] **)**	149,898	273,167	1,045,253	941,477	587,539	619,189	192,000	3,808,523
**Adolescents**	**Projection period**	2008–12	2013–17	2018–22	2023–27	2028–32	2033–37	2038–42	—
	**Averted infections**	31	66	293	305	221	270	97	1283
	**Savings US$**	404,550	861,300	3,823,650	3,980,250	2,884,050	3,523,500	1,265,850	16,743,150
	**Discounted averted infections**	29	54	205	185	115	122	38	748
	**Discounted savings US$**	232,602	423,883	1,621,957	1,460,924	911,705	960,818	297,933	5,909,820
**Adults**	**Projection period**	—	—	—	2008–12	2013–17	2018–22	2023–27	—
	**Averted infections**	—	—	—	294	213	260	93	859
	**Savings US$**	—	—	—	3,836,700	2,779,650	3,393,000	1,213,650	11,209,950
	**Discounted averted infections**	—	—	—	277	173	182	56	689
	**Discounted savings US$**	—	—	—	2,189,564	1,366,214	1,439,811	446,460	5,442,049

## Results

### Cost-Effectiveness Results for Infants, Adolescents, and Adult MC

**Figure 1:**
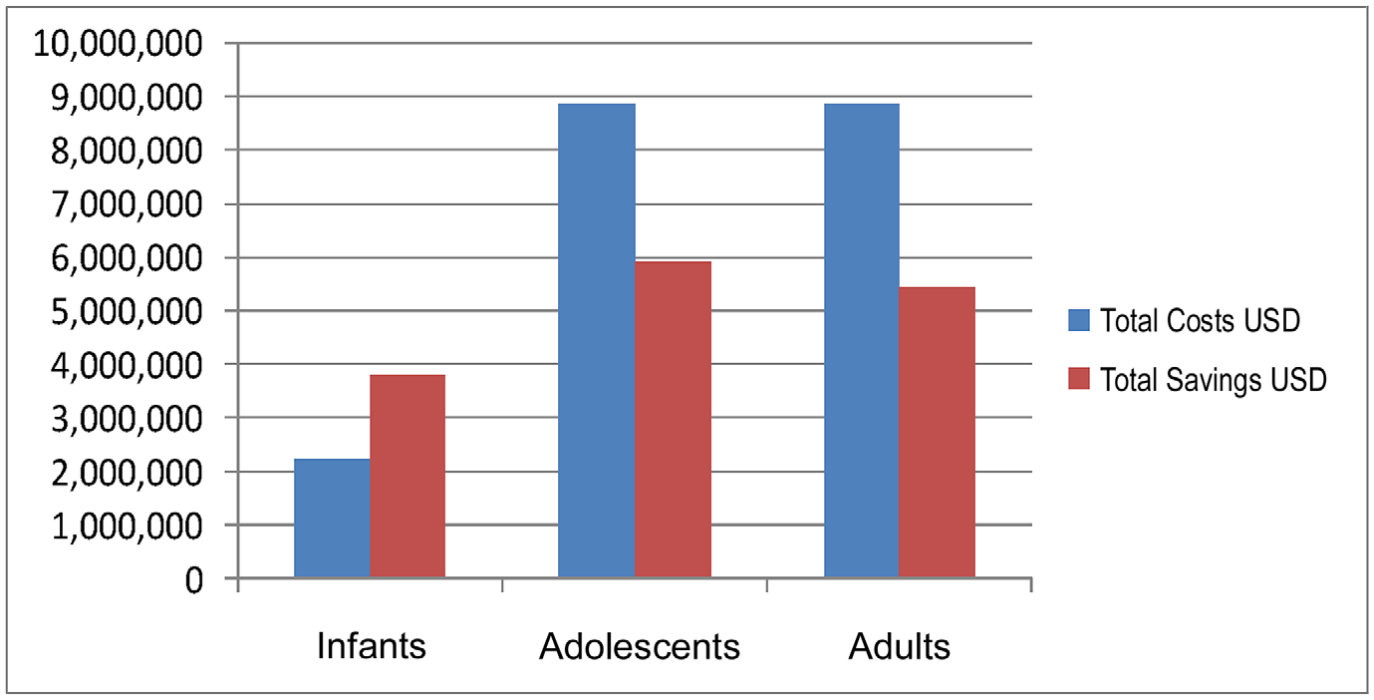
Total costs and savings for neonatal, adolescent, and adult MC (cohort 150,000 people), Rwanda, 2008.

Figure 1 provides total costs (unit cost of MC and HIV testing and counselling ×150,000) and discounted savings for the Government of Rwanda if a cohort of 150,000 people were to be tested for HIV and circumcised in 2008.

For infant MC, total costs (US$2,250,000) are lower than discounted total savings (US$3,808,523). Therefore, the intervention is cost-saving. For adolescents and adults, total costs (US$8,850,000) are higher than total savings, for net costs of US$2,940,180 and US$3,407,951, respectively. The cost-effectiveness ratio (net cost per infection averted) is US$3,932 and US$4,949 for adolescents and adults, respectively.

The findings from the analysis in Rwanda show that neonatal MC is less expensive than adolescent and adult MC (US$15 instead of US$59 per procedure) and is cost-saving; even though savings from infant circumcision will be realized later in time. The fact that MC of infants in Rwanda is a cost-saving intervention means that for each MC performed, the government of Rwanda will save money.

Still, the costs per infection averted for adolescent and adult MC are both competitive with other HIV prevention interventions. Net costs for adolescents are lower than for adults since circumcising adolescents will avert a greater number of infections than circumcising older men (the protection by MC applying to a greater number of years of exposure to HIV). Also, circumcising adolescents has the potential to avert the highest number of discounted HIV infections, because the delay from birth to initiation of sexual activity devalues the infections prevented from infant MC more than the ones prevented by adolescent MC.

The discounted cost per life year gained for adolescents and adults is US$334 and US$613, respectively. To calculate the number of life years gained, we multiplied the discounted HIV infection averted at different ages by the life expectancy at that age group (source: WHO Life Tables, 2006 [17]) less the years of survival that would have in any case occurred with HIV and treatment availability (8 y plus 14 = 22 y).

According to the WHO, the per-capita GDP, adjusted for the purchase power parity of the country, can be used for setting thresholds for cost-effectiveness. Thus, interventions for which the additional cost incurred to gain one quality-adjusted life year is less than the country's per-capita GDP are considered as very cost-effective. Considering that GDP per capita in Rwanda (based on 2007 estimates) is US$355 [27], MC for adolescents is slightly less than one GDP/per capita/life year gained and therefore highly cost-effective, while MC for adults is less than two GDP/per capita/life year gained and therefore potentially cost-effective (WHO criteria). The WHO criteria were designed to be used with disability adjusted life years, which would increase the cost-effectiveness ratios of adolescent and adult MC estimates here somewhat were that adjustment to be made.

Although the extent of monetary savings are not comparable to those associated with preventing HIV, we estimated that per each year-cohort (150,000 persons) there will be 5,000 fewer cases of syphilis and virtually no cases of penile cancer (one to two cases fewer). For children, there will be at least 2,500 fewer urinary tract infections. This finding further suggests that the estimates presented here underestimate the cost-effectiveness of the procedure.

### Sensitivity and Thresholds Analysis

Given the uncertainty embedded in the input values of the base case scenario, we conducted a one-way sensitivity analysis and explored a wide rage of values in order to identify thresholds (Table 5). We report the threshold at which the procedure costs more than one GDP/capita/life year gained (WHO criteria for cost-effectiveness). For infant MC we also report the threshold at which the intervention is no longer cost-saving.

**Table 5:** Threshold analysis.

Variable	Base Case	Cost-Saving Threshold (Infants)	Highly Cost-effective Threshold (<1GDP/Capita/Life Year Gained) GDP per Capita = US$355 (2007 Estimate) (Infants)	Highly Cost-effective Threshold (<1GDP/Capita/Life Year Gained) (Adolescents)
**Discount rate**	3%	4.1%	5.4%	3.1%
**HIV incidence**	Stable at 2008 values	40% decrease	61% decrease	2% decrease
**Protective effect of MC**	55%	33%	22%	54%
**Cost of MC procedure**	US$15 infant; US$59 adult/adolescent	US$25	US$38.50	US$60
**Cost of treatment and care**	US$14,500 per person lifetime	US$8,600	US$900	US$14,100
**Adherence to treatment**	90%	54%	6%	88%

Overall, results for infant MC appear robust. However, MC for infants is no longer cost-saving for a small increase of the discount rate. Infant MC remains highly cost-effective across a reasonable range of changes in the base case scenario.

To take into account the positive effects of the numerous HIV prevention interventions ongoing in the country, we performed a sensitivity analysis reducing the annual rate of HIV incidence from the 2008 base case (without changing other values). We found that the cost-effectiveness of neonatal MC is not very sensitive to a decrease in HIV incidence, and that neonatal MC remains cost-saving until incidence decreases 40% from the base case value, suggesting its suitability for countries with lower HIV incidence/prevalence.

Circumcision, and in particular adult MC, carries the potential for risk compensation by an increase in risky sexual behaviour. Although recent studies do not support this theory [1], we tested our results by reducing the net protective effect of MC. Neonatal MC is cost saving until a protective effect of MC of 33%, and overall results for neonatal MC are relatively insensitive to a decrease in the protective effect of MC.

Neonatal MC is cost saving up to a cost per procedure of US$25. Understandably, since benefits will happen later in life, for neonatal MC to remain cost saving the maximum cost per procedure has to be lower than the cost per adolescent and adult MC. Neonatal MC would still be highly cost-effective if the lifetime cost of treatment and care (savings per infection averted) fell to US$900. Because costs per MC are lower in infants, cost-effectiveness of neonatal MC is expected to be relatively insensitive to reduction in savings from averted treatment and care. The sensitivity to decreases in the adherence rate to ART (or to increased losses to follow-up) mirrors that for cost of treatment and care. Adolescent MC is highly cost-effective for the base case scenario but no longer so for very small changes in the input variables. Adult MC is neither cost-saving nor highly cost-effective in Rwanda when considering only the direct benefit of reduced health care costs in the circumcised man.

## Discussion

Infant MC can lower health system costs because of moderate implementation costs, high and durable protective effects, and the averted HIV-care costs. As the sensitivity analysis shows, these findings are robust across a wide range of input values for Rwanda. The study shows that adolescent MC may be a highly cost-effective intervention. MC for adults is the least cost-effective of the three procedures.

Findings are generally consistent with results from other costing studies on adult MC in Lesotho [12], Swaziland [13], and with cost-effectiveness analysis of adult MC in Uganda [14] and South Africa [15], even though in these countries HIV incidence, and consequently the number of potentially averted HIV infections, is much higher. In Uganda, with an annual HIV incidence of 1.25% and a cost per adult MC of US$69 , the cost of MC (not adjusted for savings on treatment) per HIV infection averted was estimated at US$1,485 (including the indirect effect on women). The study in South Africa shows that MC generates large net savings after adjustment for averted HIV medical costs. With an annual HIV incidence of 3.8% and a cost per adult MC of US$55.7, the cost of MC (unadjusted for averted medical care costs) per HIV infection averted was estimated at US$181 (including the indirect effect on women); while for 1,000 circumcisions net savings (adjusted for averted medical care cost) were US$2.4 million.

A recent study by White et al. [16] also found that MC is a cost-saving intervention in a wide range of scenarios of HIV and baseline circumcision prevalence. The authors predict that circumcising neonates, although cheaper, would only become cost-saving after around 30 y (within the time horizon of our study). These findings are consistent with ours because our model considers the net present value of the interventions, extended to the entire life of the circumcised individuals. The absolute cost per infection averted is significantly lower in the White paper than in ours, but this is to be expected given that they estimated benefits of reduced secondary infections among the sexual partners of the circumcised men, while we did not. The White paper also concludes that as neonate and adult programmes are likely to be relatively noncompetitive for staff, facilities, and training, an optimal strategy may be to scale up both simultaneously, which is also consistent with our findings, albeit for a setting with much lower incidence and prevalence.

Neonatal MC is a less expensive procedure (faster, less complicated, and with fewer side effects) than adolescent and adult circumcision and can also be cost-saving (even when considering the discounting effect). Most importantly, infant circumcision can be easily integrated into existing health services (such as neonatal visits and vaccination sessions) and, where health workers are well trained, it does not require skilled surgeons and parallel structures that could drain an already weak system. Moreover, neonatal MC may carry less risk of a compensatory increase in risky sexual behaviour and it is likely to be more protective than adolescent or adult circumcision because there is no possibility of sexual activity during healing. Finally, circumcision among children does not carry the same implications as those for adolescents and adult MC, such as discomfort, stigma, and days out of school and work (with their associated opportunity costs).

We deduce that infant circumcision has a better potential to achieve the very high coverage over time of the population required to achieve maximal reduction on HIV incidence than adolescent and adult circumcision.

This model assumes similar sized cohorts for adolescents and adults as for infants, and one might wonder why, given that the population in need of circumcision is so much larger than a single birth cohort. This was done for several reasons. First, the government policy question that prompted the study was whether infant circumcision should be added as a strategy to that already proposed for adolescents and adults. Thus, by using the size of the Rwandan birth cohort, the per-person costs could be compared with MC at other ages and the total costs and affordability of infant MC could also be assessed. Since the model does not attempt to estimate secondary benefits (e.g., to the female partners of circumcised men) or the herd effect of high levels of MC, the relative results will be the same regardless of whether the model is run with a cohort of one or 150,000. However, while 150,000 children represent a high level of annual coverage of the birth cohort, a realistic strategy for adolescent or adult catch-up should probably aim for higher annual coverage. A realistic assessment of what coverage levels could be attained, and at what cost (especially one that considered effects of scale on program costs) goes beyond the purpose of this study.

Given that this study does not quantify the indirect benefits of MC, the cost-effectiveness estimates are conservative. This is likely to be even truer for infants than for adolescents and adults for two reasons: MC coverage of infants is likely to be much higher, potentiating the herd effect, and, behavioural compensation is less likely to occur with infants.

Any modelling exercise is at best an approximation of reality. Studies that model the future, like this one, approximate a reality that does not yet exist, and this requires making a number of assumptions about the future (for instance on what will happen to HIV incidence rates, on the effectiveness of large-scale circumcision, and on the costs of HIV treatment in the future). Thus, these results, like those of similar exercises, must be seen as valuable inputs into decision-making because they identify likely impacts of different courses of action. They cannot pretend to eliminate the uncertainty that underlies such decisions, just to reduce it. As mentioned above, this model also has limitations related to what it does and does not include. The most important of these limitations is the fact that the model only takes the prevention benefit for the circumcised individual into consideration, and not for his sexual partners and offspring.

### Conclusions

In this study we show that MC for infants is not only highly cost-effective but also likely to be cost-saving and that MC for adolescents is a cost-effective procedure. Although benefits will be gained later in life, these positive results are stronger for circumcision of male newborns. The next step for Rwanda is to explore how best to introduce MC at different ages, including an appropriate mass media campaign. A pilot implementation exercise in one district, accompanied by close monitoring and operational research on key variables, should be followed by a scale-up of the program country wide.

Given the low cost and long term benefits, this study suggests that countries with moderate HIV epidemics should offer routine infant circumcision, integrated into existing health services. In addition, adolescents should be offered MC until aging of circumcised infants renders it obsolete and adult MC should be offered with priority to population groups with a high level of HIV incidence. Owing to the increased complexity of this strategy, each country will need to consider a variety of options to achieve high levels of coverage with adolescent/adult MC. Options may include specialized centres, mobile surgery units, and specialized surgery teams that move from clinic to clinic. MC should be offered as part of an integrated HIV prevention package that includes promotion of safer sex (delayed initiation, reduction in multiple/concurrent partners, and access to condoms).

African leaders and development partners should stop managing the HIV response as only an emergency issue and release themselves from a 1-y or even a 5-y planning perspective to focus on sustainable long-term choices for countries. From a development perspective, because infant MC is proven to be an effective means of HIV prevention, action cannot be deferred simply because gains will be in the distant future. National plans should be made accordingly on the basis of the best available information, knowing that currently there is neither a vaccine nor cure for AIDS, while remaining open and flexible to adaptation if better solutions arise. In the presence of infant MC, adolescent and adult MC would evolve into a “catch-up” campaign that would be needed at the start of the program but would eventually become superfluous upon attainment of high levels of infant coverage. Infant circumcision is likely to be highly cost-effective even in countries with lower incidence than Rwanda. In Rwanda, if the dynamic benefits of circumcision (prevention of secondary infections) are considered in addition to the health benefits for the circumcised man, even adult MC is likely to be close to or below the highly cost-effective threshold. This finding suggests that Rwanda should be simultaneously scaling up circumcision across a broad range of age groups, with high priority to the very young.

## Abbreviations

ART: antiretroviral therapy
CAMERWA: Central Purchasing of Essential Medicines in Rwanda
CI: confidence interval
GDP: gross domestic product
MC: male circumcision
MOH: Ministry of Health
OI: opportunistic infection
PMTCT: prevention of mother to child transmission
RNIS: Rwanda National Institute of Statistics
RR: risk ratio
STI: sexually transmitted infection

## References

[1] Joint United Nations Programme on HIV/AIDS (UNAIDS) and World Health Organization (WHO) (2007). Male circumcision: global trends and determinants of prevalence, safety and acceptability.

[2] Department of Statistics, in the Ministry of Economics (MINECOFIN) and MEASURE DHS Interim Rwanda Demographic and Health Survey (2007–2008).

[3] Gray RH, Kigozi G, Serwadda D, Makumbi F, Watya S (2007). Male circumcision for HIV prevention in men in Rakai, Uganda: a randomized controlled trial.. Lancet.

[4] Bailey RC, Moses S, Parker CB, Agot K, Maclean I (2007). Male circumcision for HIV prevention in young men in Kisumu, Kenya: a randomized controlled trial.. Lancet.

[5] Auvert B, Taljaard D, Lagarde E, Sobngwi-Tambekou J, Sitta R (2005). Randomized, controlled intervention trial of male circumcision for reduction of HIV infection risk: the ANRS 1265 trial.. PLoS Med.

[6] Weiss HA, Thomas SL, Munabi SK, Hayes RJ (2006). Male circumcision and risk of syphilis, chancroid and genital herpes: a systematic review and meta-analysis.. Sex Transm Inf.

[7] Tobias AR, Serwadda D, Quinn TC, Kigozi G, Cravitt PE (2009). Male circumcision for the prevention of HSV-2 and HPV infections and syphilis.. N Engl J Med.

[8] Singh-Grewal D, Macdessi J, Craig J (2005). Circumcision for the prevention of urinary tract infection in boys: a systematic review of randomised trials and observational studies.. Arch Dis Child.

[9] WHO, UNAIDS, UNFPA, UNICEF, The World Bank (2007). Information package on male circumcision and HIV prevention. Insert 3.

[10] WHO, UNAIDS, JHPIEGO (2007). Manual for male circumcision under local anaesthesia. Version 2.5B.

[11] Department of Statistics, in the Ministry of Economics (MINECOFIN) and MEASURE DHS (2005). Rwanda demographic and health survey III.

[12] USAID I Health Policy Initiative (2007). Costing male circumcision in Lesotho and implications for the cost-effectiveness of circumcision as an HIV intervention.

[13] USAID I Health Policy Initiative (2007). Costing male circumcision in Swaziland and implications for the cost-effectiveness of circumcision as an HIV intervention.

[14] Gray RH (2005). Reducing HIV transmission: lessons from Rakai and other African studies. International AIDS Society. Rio de Janeiro, Brazil. Reported in UNAIDS and WHO (2007) Male circumcision: global trends and determinants of prevalence, safety and acceptability.

[15] Kahn JG, Marseille E, Auvert B (2006). Cost-effectiveness of male circumcision for HIV prevention in a South African setting.. PLoS Med.

[16] White RG, Glynn JR, Orroth KK (2008). Male circumcision for HIV prevention in sub-Saharan Africa: who, what and when?. AIDS.

[17] WHO (2006). Life tables.. http://www.who.int/whosis.

[18] UNAIDS (2007). Safe, voluntary, informed male circumcision and comprehensive HIV Prevention: programming guidance for decision-makers on human rights, ethical and legal considerations.. http://data.unaids.org/pub/Manual/2007/070613_humanrightsethicallegalguidance_en.pdf.

[19] CNLS, TRAC Plus, NIS, MOH, UNAIDS, WHO, USAID/Health Policy Initiative (2008). HIV-AIDS in Rwanda. 2008 Epidemic update.. http://www.cnls.gov.rw.

[20] World Health Organization (2002). The world health report, 2002 - Reducing risks, promoting healthy life. Technical considerations for cost-effectiveness analysis. Chapter 5.

[21] UNAIDS (2009). http://www.unaids.org/en/KnowledgeCentre/HIVData/Epidemiology/.

[22] Walensky RP, Paltiel AD, Losina E, Mercincavage LM, Schackman BR (2006). The survival benefits of AIDS treatment in the United States.. J Infect Dis.

[23] NACC/CNLS (2007). Dataset to estimate the amount of financial resources needed to implement the National Strategic Plan on HIV (2005–2009). An application of the Goal and Resource Needs Model, Rwanda.

[24] CNLS Plan National de Prevention 2005–2009, VCT unit cost (Annex 4).

[25] WHO/INSP global costing exercise for Sub-Saharan African and Agence Européenne pour le Développement et la Santé (AEDS), 2005..

[26] TRAC (2007). Report on the evaluation of clinical and immunologic outcomes from the national antiretroviral treatment program in Rwanda, 2004–2005..

[27] International Monetary Fund (2008). World Economic Outlook Database.

